# Electronic health record data quality variability across a multistate clinical research network

**DOI:** 10.1017/cts.2023.548

**Published:** 2023-05-15

**Authors:** Yahia Mohamed, Xing Song, Tamara M. McMahon, Suman Sahil, Meredith Zozus, Zhan Wang, Greater Plains Collaborative, Lemuel R. Waitman

**Affiliations:** 1 University of Missouri-Kansas City School of Medicine, Kansas City, MO, USA; 2 University of Missouri School of Medicine, Columbia, MO, USA; 3 University of Texas Health Science Center at San Antonio, San Antonio, TX, USA; 4 Collaborators

**Keywords:** Electronic health records, data quality, PCORnet, common data model, Greater Plains Collaborative

## Abstract

**Background:**

Electronic health record (EHR) data have many quality problems that may affect the outcome of research results and decision support systems. Many methods have been used to evaluate EHR data quality. However, there has yet to be a consensus on the best practice. We used a rule-based approach to assess the variability of EHR data quality across multiple healthcare systems.

**Methods:**

To quantify data quality concerns across healthcare systems in a PCORnet Clinical Research Network, we used a previously tested rule-based framework tailored to the PCORnet Common Data Model to perform data quality assessment at 13 clinical sites across eight states. Results were compared with the current PCORnet data curation process to explore the differences between both methods. Additional analyses of testosterone therapy prescribing were used to explore clinical care variability and quality.

**Results:**

The framework detected discrepancies across sites, revealing evident data quality variability between sites. The detailed requirements encoded the rules captured additional data errors with a specificity that aids in remediation of technical errors compared to the current PCORnet data curation process. Other rules designed to detect logical and clinical inconsistencies may also support clinical care variability and quality programs.

**Conclusion:**

Rule-based EHR data quality methods quantify significant discrepancies across all sites. Medication and laboratory sources are causes of data errors.

## Introduction

Electronic health records (EHR) adoption rapidly increased since Congress passed the Health Information Technology for Economic and Clinical Health (HITECH) Act in 2009 [1]. By 2021, certified EHR adoption reached 78% for office-based physicians and 96% for nonfederal acute care hospitals [2]; resulting in a substantial increase in the capture patient health-related information available for learning health systems and the national health data infrastructure [1]. Widespread EHR adoption increased interest in using the real-world data to support clinical research such as pragmatic clinical trials, comparative effectiveness studies, observational studies, and safety surveillance studies [3]. Many federal agencies, such as the Food and Drug Administration (FDA) and the National Institute of Health (NIH), have increasingly encouraged the use of EHR data to advance clinical research [4]. However, there are many concerns about fitness of EHR data to conduct clinical research and support clinical decision support systems [5,6]. The complexity of aggregation, acquisition, processing of EHR data to be ready for secondary use and harmonizing data from different clinical sites create many quality issues that may impact the validity of research results and affect stakeholders’ decisions [3,5]. In 2021, the FDA published draft guidance for real-world data use and recommended researchers to examine the data using a rigorous approach to assess completeness, accuracy, and plausibility. Researchers are encouraged to provide a rationale for using a specific methods to evaluate these data quality dimensions and describe their application in the study protocols [7].

Various methods have been used to assess EHR data quality, but the field has yet to reach consensus. Weiskopf and Weng [8] described the standard methods used to assess the data quality, such as comparing to a gold standard, data element agreement, element presence, data source agreement, distribution comparison, validity checks, and log review. They recommended adopting a valid and systematic approach to assess EHR data quality. Rule-based approaches for EHR data quality assessment have been used previously by many researchers who proved their effectiveness in identifying data discrepancies. Carlson *et al*. [9] developed rules to assess the quality of data used to calculate decision support scores because they found some critical data they needed in producing the scores were unavailable, out of range, or inconsistent. The results showed data become more accurate after the implementation of rules. Brown and Warmington [10] described a method based on the rules approach to identify the data error and monitor EHR data quality. The method utilized the principle of data quality probes (DQP) that increase the clinician’s focus during data entry and help them receive timely feedback on their performance. They found that DQP helped track data quality and clinical care quality over time. Kahn *et al*. [11] proposed a conceptual model that used five categories of rules to address data variability at different sites. Hart and Kuo [12] built a rule-based system at Island Health to assess the quality of EHR data. The system could identify errors related to EHR data or the system itself. This helps the team to address these errors. Wang *et al*. [13,14] used Kahn’s conceptual model to develop a framework that includes rule templates and knowledge tables and used structural query language (SQL) to implement them against the EHR database of a single center and validate by discussing his results with clinical experts and reported their feedback.

The Greater Plains Collaborative (GPC) [15,16] is one of the Patient-Centered Outcomes Research Network (PCORnet) Clinical Data Research Network (CDRN), which includes 13 medical centers across eight states. The total population in the GPC network is over 34 million patients. The GPC Reusable Observable Unified Study Environment (GROUSE) unified EHR data with claims data from the Center for Medicare and Medicaid Services (CMS) [17]. This makes GPC a comprehensive data source for conducting clinical research using healthcare and insurance claims data. Currently, PCORnet coordinating center uses a structural approach to check for data integrity and characteristics called empirical data curation. The results of the data curation are summarized in the empirical data characterization (EDC) report and shared with the network partner which is reported quarterly. For data integrity, EDC report includes 13 rules to check for model conformance, plausibility, completeness, and persistence [18]. However, these rules do not cover granular information such as range or unit of laboratory values, drug interaction, and laboratory test orders related to medication prescription. This limits the translation of the findings from the EDC report to the specific needs of clinical research and health learning system initiatives.

In a previous study, we tailored the rules in Wang’s framework to be compatible to run against the PCORnet common data model [19]. The rules were implemented to assess the quality of EHR data from the University of Missouri Health Care System, GPC’s coordinating center. In this study, we expand the previous work to (1) assess the variability of EHR data quality cross multiple healthcare systems within GPC, (2) examine ability of rules to track clinical care quality, (3) compare the results with the PCORnet EDC report, and (4) report on the scalability of the framework.

## Materials and Methods

### Data Source

We used the rule-based method [19] to assess the EHR data quality of 13 clinical sites that contributed to GROUSE. We requested data from the clinical sites according to the institutional review board (IRB) protocol and received approval from all sites willing to participate in the study. GROUSE’s established IRB protocol aims and data sharing agreement and provided de-identified data from all sites [17]. The subsequent use of deidentified data for quality assessment was determined as nonhuman subject research. The data were stored following the PCORnet CDM V6.0 specification on the deidentified instance on the Snowflake cloud data platform [20]. The name of sites deidentified using alphabetic order (A-M) to preserve anonymity.

### Design of the Prior Framework

Wang *et al*. [13,14] formulated the framework used in this study by reusing sets of rules published by many sources such as OHDSI, PCORnet, and Sentinel networks. To identify new rules to extend the framework, they engaged stakeholders and clinicians in system design and validation of additional new rules. The framework component includes 1) knowledge tables which include the information needed to implement the rules against the databases and 2) rule templates, which assess for value out of range, incompatibility, incompleteness, a temporal sequence error, and duplication. A total of 63,397 rules in 28 templates were categorized based on Kahn’s conceptual model and corresponded with the recently proposed Harmonized Data Quality Assessment Terminology (conformance, completeness, and plausibility) [21].

### Implementation of Rules on Different Clinical Sites in GROUSE

We loaded the knowledge tables into GROUSE along with implemented rules structured query language (SQL) queries described in our prior study [19] for execution against common data model tables from participating sites. Discrepancies were identified and recorded at the patient and encounter level. A discrepancy is defined as an instance of one or more data values that does not match the values in the knowledge table of a specified rule. Therefore, the rule will flag any instance that has data value inconsistent with the value in the knowledge table. We counted the discrepancies at the encounter and patient levels and calculated the percentage of discrepancies with discrepancy number as the numerator and the total number of encounters or patients as the denominator for each rule template. The variability between sites (A-M) was assessed by comparing the percentage numbers after calculating the percentage of discrepancy at both encounter and patient levels and plotting the results for visualizations.

The rule templates were categorized to assess five data quality dimensions:

- Value out of range: The rules in this category will check and detect any value out of data constraints. For instance, weight value above 300 kg or below 1 kg.
- Incompatibility: Rule templates in this category will assess the relational and attribute dependency and locate any inconsistent instances. For example, rules that check for gender and procedure will flag any record with a female gender and prostatectomy procedure.
- Incompleteness: Rule templates in this category assess the multivariate and record-level missingness or presence of data elements. Rules in one template check for the presence of laboratory test monitoring for some prescribed medication. For example, a lithium prescription needs blood level monitoring to avoid lithium toxicity.
- Date and time error: Rule templates in this category assess the chronological and temporal relationship among data elements. For example, rules in the laboratory time template checked for chronological relationships between the type of laboratory test and the specimen collection time to provide accurate results for the test.
- Duplication: Rule templates in this category assess record duplication for procedures performed once in life. For example, rules will determine if data have duplicated records for hysterectomy or prostatectomy procedures.

### The Ability of Rule Templates to Monitor Data Quality and Track Clinical Care Quality

Some rules in the framework were based upon clinical scenarios though these rules will fire to any inconsistency. We took advantage of this characteristic and conducted follow-up analyses to determine whether the data discrepancies captured by these rules were due to actual transformation issues, variability in clinical care, or quality of care issues. We used the rule template drug and laboratory to check for ordering prostate-specific antigen (PSA) tests before or after initiation of testosterone replacement therapy (TRT). We looked at male patients who were 40 or above and had any prescription of testosterone cypionate 200 mg/ml injectable solution and if there was any order for PSA in their records.

### Comparison of Results with EDC Report

The current data quality assessment implemented by the PCORnet Coordinating Center’s EDC report examines a) data model conformance to assess the value of constraints, b) data plausibility to assess future dates, illogical dates, encounters per visit and per patient, C) completeness to assess diagnosis records per encounter, procedure records per encounter, missing or unknown values, and laboratory result data, and C) data persistence to assess table changes, selected encounters, or code types. However, all these rules assess only structural data quality issues but not sematic data quality issues revealing more detailed information and looking deeper into potential root causes. For example, the type of unit used for laboratory tests or specific physiologically acceptable value range, incompatibility of diagnosis with gender or age, incompatibility of the procedure with gender or age, and check for drug interaction of prescribed medication. We will use the results generated by the rules framework to compare with the results published in the EDC report for every site.

### Scalability of the Framework and Storing the Output of Data Quality Assessments

We benchmarked all SQL queries after the implementation of rules, identified the rules with longer execution time, and compared that time in milliseconds between rule templates that need a simple query to implement versus the complex one. We created a schema on the GROUSE database to store all results from implementing rules across sites to assess data quality improvement and scalability across sites after each quarterly data refresh cycle.

## Results

For this study, we focused on 17 logic templates that included 8199 rules and successfully executed them against the common data model (CDM) of 13 sites on GROUSE. Most sites showed similar discrepancies patterns and performed well in many data quality dimensions examined by rules templates. However, we still identified several sites with significant discrepancies. Supplementary Table 1 summarized the number and percentage of discrepancies at the patient level, and Supplementary Table 2 summarized the number and percentage of discrepancies at encounter levels for every site.

### Assessment of Conformance (Out of Range Values)

**Figure 1.**
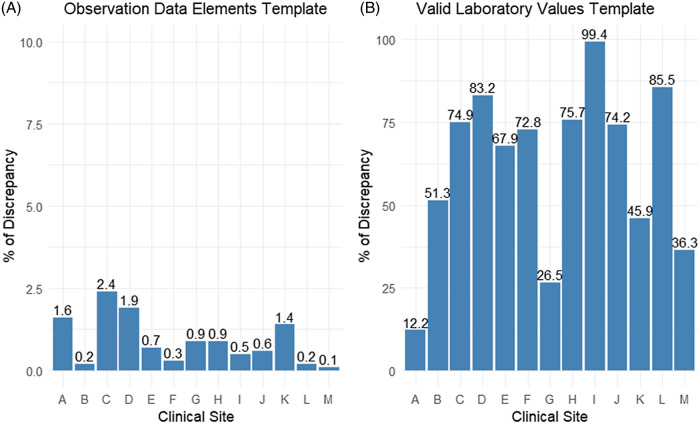
Rule templates assessing out of range values in all sites.

Three templates including 43 rules were used to assess the data conformance at each site. The demographic data elements template checked for records with a date of birth before 01/01/1850, which revealed 0% discrepancies in all sites. The observation data element template assessed the height, weight, and diastolic and systolic blood pressure values using the vital table, showing variation in the discrepancy percentage among sites. The analysis showed site C had a 2.4% discrepancy, followed by site D, which had a 1.9 % of discrepancy. Sites A and K showed a 1.6% and 1.4 % discrepancy, respectively. Other sites kept the discrepancy percentage below 1% (Fig. 1A). Most of these discrepancies were due to populating fields for height, weight, and diastolic/systolic blood pressure measurements with extremely small or large values and null values. The valid laboratory values template, which assessed a group of laboratory normal range values and units, showed discrepancies which were more than 10 % of patients in all sites, with the greatest discrepancy in site I which reached 99.4% (Fig. 1B). These discrepancies were due to the use of measurement units for laboratory tests different from the Logical Observation Identifiers Name and Codes (LOINC) units, and some sites have populated units with no information (NI) or NULL values.

### Assessment of Consistency (Incompatibility)

**Figure 2.**
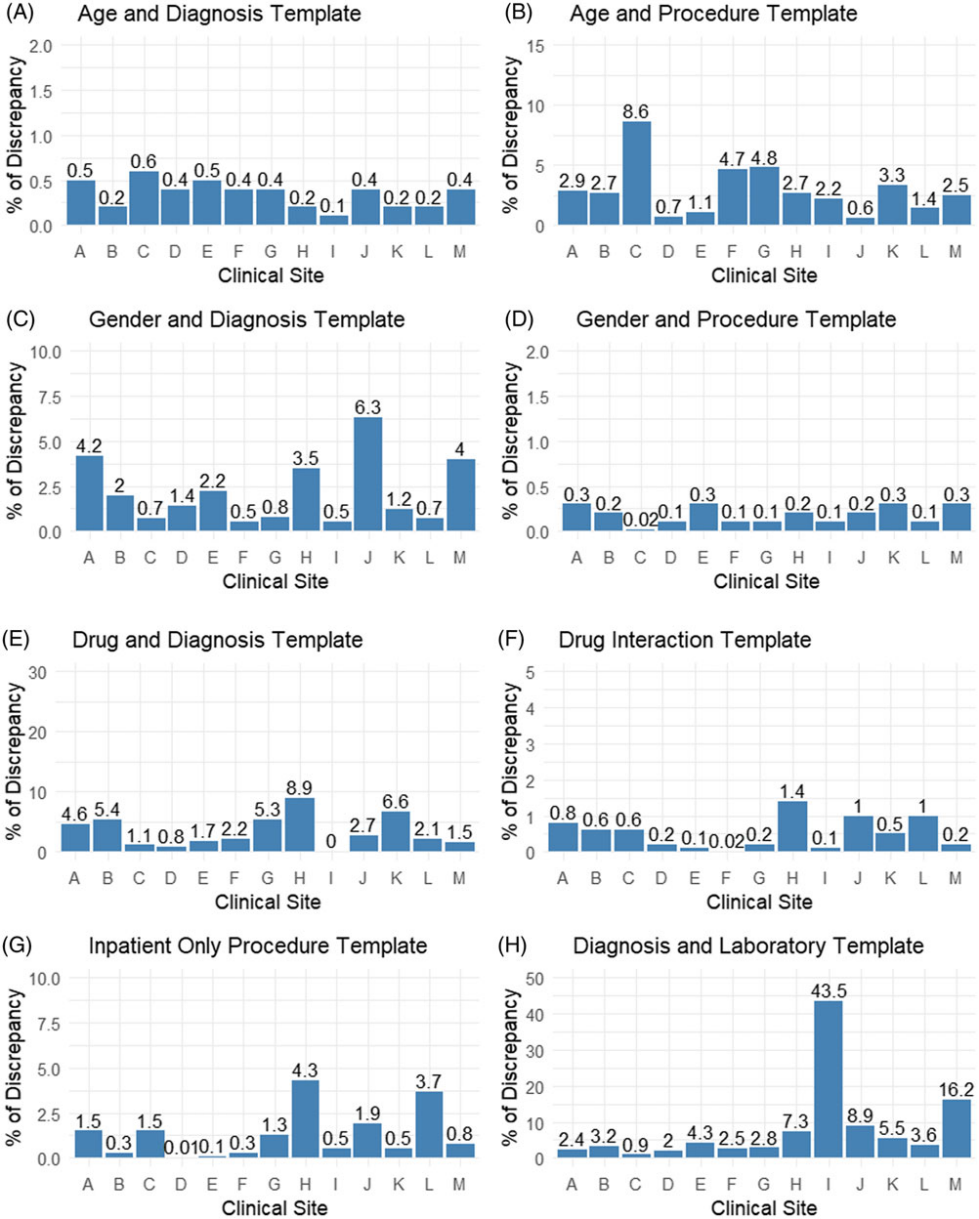
Rule templates assessing incompatibility in all sites.

The incompatibility of data values was verified by executing a set of rules that include 8,112 rules categorized into nine templates as follows. The rules in the template of age and diagnosis assessed records that have inconsistent age with disease diagnosis and showed less than 1% of the patients have incompatible age with the recorded diagnosis in all sites (Fig. 2A). Most of these discrepancies were due to mapping the diagnosis of a fussy infant (baby) to the age of 1 year. The rules in the age and procedure template checked for patient age inconsistency and performed procedures. The results revealed site C had 8.6% of patients with age incompatible with the procedure, whereas the discrepancy dropped to less than 5 % of patients in the other sites (Fig. 2B). The discrepancies captured by this rule were due to the periodic comprehensive preventive medicine reevaluation, and preventive visits for an established patient were mapped incorrectly to the specified age. The rules in the template of gender and diagnosis checked the consistency between the gender of patients and the recorded diagnosis. The results depicted that site J had 6.3 % of the patient’s gender was incompatible with the recorded diagnosis. The other sites showed that less than 5% of the patients had a gender incompatible with the recorded diagnosis (Fig. 2C). Most of the discrepancies were due to encounters with a diagnosis of single live birth that were mapped to the male gender because they attributed the gender of the born baby instead of the mother’s gender. Rules in the gender and procedure template assessed the consistency of the patient’s gender with the recorded procedure. The results showed all sites had less than 1% of their patients had incompatible ages and procedures (Fig. 2D). Most discrepancies were due to incorrectly mapping the obstetric panel and urine pregnancy test to the male gender.

Rules in the drug and diagnosis template assessed the possibility of in-hospital medication prescription that should not be prescribed for a disease specified by the rules on the same date of disease diagnosis. The results showed sites H, *K*, B, and G had the greatest discrepancy percentage, which is 8.9%, 6.6%,5.4%, and 5.3%, respectively. All other sites showed a percentage below 5% (Fig. 2E). The discrepancies were due to the prescription orders for non-steroidal anti-inflammatory drugs (NSAIDs) were on the same date of diagnosis with peptic ulcer disease. Rules in the drug interaction template assessed the data for prescribing two medications with known drug interaction within the same encounter date. Our results revealed that only site H had 1.4% discrepancy. The rest of the sites had 1% or less (Fig. 2F). Discrepancies captured by this rule template were primarily due to the prescription of potassium chloride on the same date as prescribing spironolactone or clonidine and propranolol on the same date. The rules in the inpatient-only procedure template checked for any procedure that should be performed in the hospital but was recorded as outpatient or ambulatory visit. Major joint replacement procedures, spinal surgery, and cesarean delivery were the most discrepancies seen in different sites and were incorrectly mapped to ambulatory visit encounters. The results showed sites H and L revealed 4.3% and 3.7% discrepancy, respectively. Other sites kept the percentage of discrepancy below 2% (Fig. 2G). The rules in diagnosis and the laboratory template assessed the consistency of the presence of laboratory tests that should be ordered for the specified diagnosis in the rules. Our results showed that sites I, M, J, H, and K had 43.5 %, 16.2%, 8.9%, 7.3%, and 5.5% discrepancies, respectively. The remaining sites showed a percentage of discrepancy below 5% (Fig. 2H). The missing LOINC codes in the laboratory table were one of the problems that led to these discrepancies.

### Assessment of Data Element and Value Completeness (Incompleteness)

**Figure 3.**
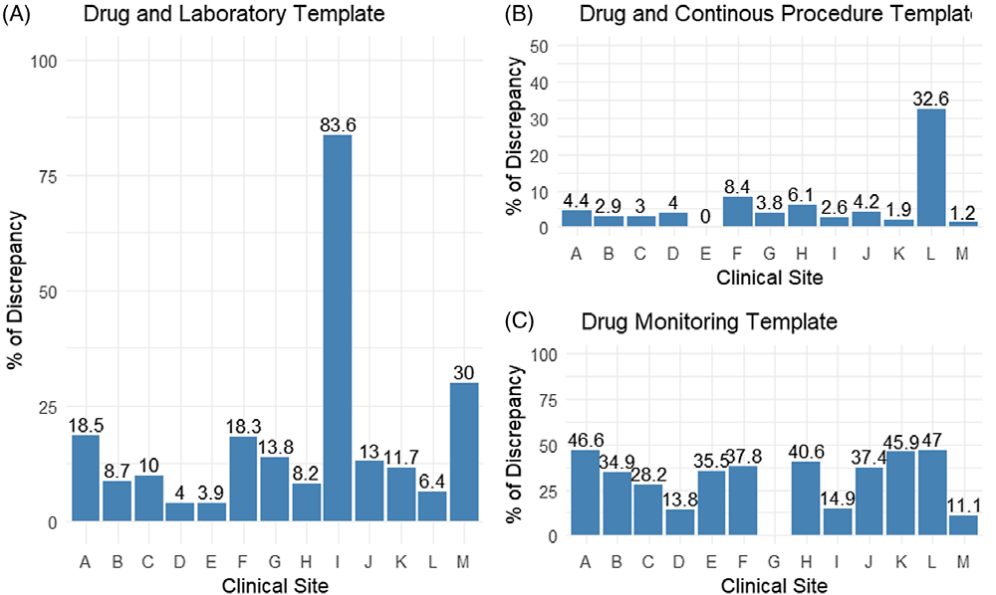
Rule templates assessing the incompleteness in all sites.

We assessed the presence of data elements and values by implementing another set of rules that included 35 rules categorized into three templates. The analyses showed the rules in drug and laboratory templates checked for the presence of laboratory tests that need to be ordered for monitoring medication side effects. This rule showed that sites I, M, A, and F had 83.6, 30%, 18.5%, and 18.3% discrepancies, respectively. Other sites showed less than a 15% discrepancy (Fig. 3A). These discrepancies in some sites were associated with the incompleteness of LOINC codes. The rules in the drug and continuous procedure template verify the presence of a specific procedure or clinical exam that needs to be performed after a specified period of medication prescription. Our results showed sites L, F, and H had 32.6%, 8.4%, and 6.1% discrepancies, respectively. All other sites kept the percentage of discrepancy below 5% (Fig. 3B). Most discrepancies were due to the RXNorm concept-unique identifier (CUI) incompleteness. The drug monitoring template verified the presence of records for the order of specific laboratory monitoring tests for a specified medication. The results showed that all sites had a percentage of discrepancy of more than 30 % except sites C, D, I, and M, which showed a discrepancy of less than 15% (Fig. 3C). These discrepancies were associated with the incompleteness of LOINC codes and RXNorm CUI at some sites.

### Assessment of temporal relationship (Date and time)

**Figure 4.**
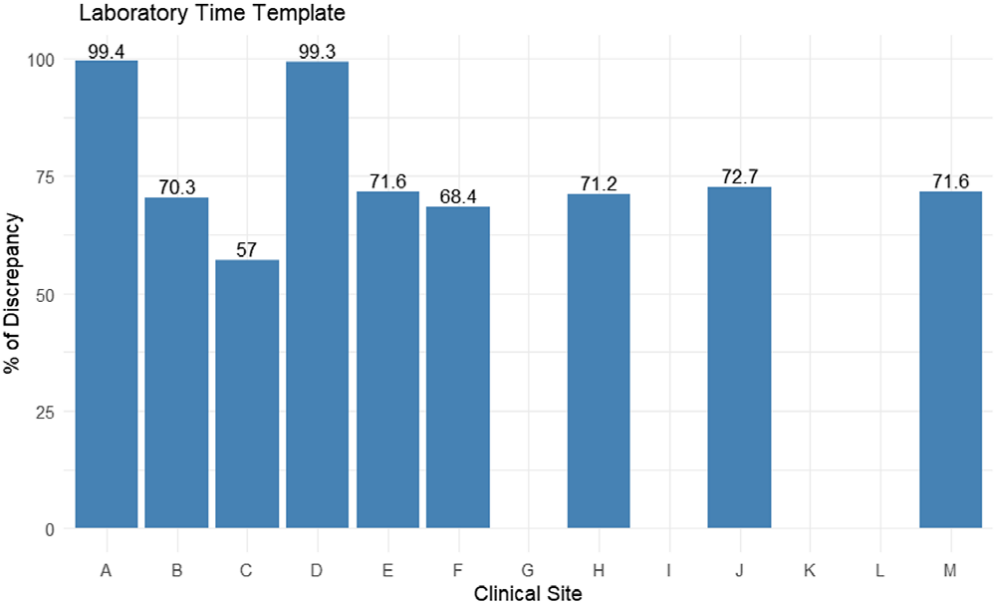
Rule template assessing the time error in all sites.

We used six rules included in two templates to verify the temporal relationship for time and date of a specific data value. After implementing the rules in the laboratory time template, all sites showed a significant discrepancy percentage of more than 50%, whereas four sites showed an empty bar chart (Fig. 4). Most discrepancies were due to the inconsistent time of specimen collection. The other rules in future date template checked for relation of dates. Most sites showed 0 or less than 0.01% discrepancy. Only site H showed 2% discrepancy in death date. The discrepancy is due to incorrectly recording death date in future years (Supplementary Table 1).

### Assessment of event duplication (Duplication)

We used three rules included in one template to check for the propensity of duplication of procedures that are impossible to occur more than once in life. Results show 0% of patients had a discrepancy in all sites.

### The ability of rule templates to capture the variability of clinical care or quality of clinical care

The laboratory and drug rule template assess data’s completeness by checking for medication prescription that should be monitored with a specified laboratory test to prevent medication side effects. We evaluated one of these rules to explore care variability in ordering PSA tests before or after starting TRT. Our results show many sites have more than 20% of patients who received TRT without any PSA test in their records. Only sites B, *C*, D, *G*, and M had below the 20% of their patients were tested for PSA (Table 1).

### Comparing the results with the EDC report

We looked at the EDC report for every site and found the report was organized to provide detailed statistical information about the data distribution and descriptive analysis. The information was included in tables and plots to simplify reading the report. In addition, it explored the assessment of different data quality dimensions. The framework used in this study provided rule templates that used clinical knowledge to capture the data errors with an intention to identify potential root causes. For example, the data of these sites passed the data curation process, and when we implemented the rule templates, our results showed rules captured many discrepancies related to different data quality problems such as incompatibility and incompleteness.

### Performance of the framework

We reviewed the history of the computation of all queries we run to implement the framework on the database for every site. It takes 11 hours and 50 minutes to run the whole script of all rule templates on 13 sites. The queries for age and diagnosis rule template were the longest, which took 25 minutes and 14 seconds to finish. Most queries for other rule templates finished in less than 15 seconds which was similar in most sites.

## Discussion

This paper expands previous work that tailored rules to operate against the PCORnet CDM [19]. We used the same framework to determine the variability of EHR data quality across 13 healthcare systems. Our results showed all sites have limited issues with demographic data, date temporal relation, or duplication of procedures. However, most sites had significant discrepancies with data related to laboratories or medications. The rules assessing laboratory and medication data based on clinical scenarios guide evaluation of plausibility or completeness of diagnosis, laboratory tests, procedures, or prescribed medications. These have the potential to heighten the clinician and health system stakeholder engagement compared with EDC reports.

The execution of valid laboratory values to assess data incompatibility also revealed a significant discrepancy in most sites. For example, site I reached 99.4% which was due to the field of the laboratory unit was 100% populated with NI (No Information). Similarly, site L showed an 85.5% discrepancy; we found that 84 % of these patients specified by the rules had records populated with laboratory units different from those specified by the rule. After grouping laboratory unit field, we found 71.4% of the field was populated with OT (Other) instead of the actual measurement unit. Thus, our findings showed most sites struggled with populating the field of laboratory units, or they used different measurement units than the LOINC unit, which is specified by the rule in addition to the null value or issue with the value range limit.

Furthermore, implementation of diagnosis and laboratory rule template that checks for patients who have a diagnosis of diabetes mellitus and any lab order for blood glucose or HbA1C test revealed a significant discrepancy in sites I and M. With further analyses, we found the LOINC code was not populated in 54.6% of patients' records for site I, which was one of the new sites joined PCORnet and GPC in 2022. In site M, we found the LOINC code was populated in 100% of patients’ records, and 16% of patients did not have any order of blood sugar or HbA1C test in their records which could be due to data entry problem or given the site’s role as a tertiary care facility, the laboratory test may have been recorded in another healthcare system and, the results returned as unstructured data.

The data completeness assessment used a drug and laboratory template that used three medications that needed follow-up with specific blood tests to monitor the side effects after the prescription. Our findings showed many sites have a significant discrepancy. For instance, site I revealed 83.5%. We found this site has much missingness in LOINC codes that reached 54.6%. For sites M, *F*, and A, there was no missingness in LOINC codes. This brought other possibilities, such as errors in data entry, the data being entered into the procedure table using CPT codes, tests performed in another healthcare system, or data recorded as unstructured data. Also, we cannot rule out possibilities of care quality issues. The drug and continuous procedure rule template checked for patients who received hydroxychloroquine prescriptions and regular eye exams every 2 year. The results showed site L has the greatest discrepancy. The analysis showed 8.8% of patients’ records missed the RXNorm CUI in the prescription table. Similarly, site F showed incomplete RXNorm CUI data reaching 17.4%. Additionally, some sites had all the data in the prescribing and procedure table and showed discrepancies, which could be due to data entry errors or procedures performed in other healthcare systems.

Moreover, the drug monitoring rule template assessed the completeness of data for some medication that needs monitoring for their level in the blood to prevent drug toxicity. The rule captured a significant discrepancy in most sites. Many sites have incomplete data for LOINC codes and RXNorm CUI, leading to these discrepancies. Whereas site G has an empty bar chart because all the LOINC codes specified by the rule template were not available in the data even though there was no missing data value in the field of LOINC codes. The time temporal relationship was assessed by the laboratory monitoring rule template, which look at the time of testosterone and salivary cortisone tests, which should be collected before 10:00 am. The results showed more than a 50% discrepancy in most sites. Many reasons led to these discrepancies, including some sites having the specimen time inconsistent with the rule of just minutes, errors in data entry, or problems in care delivery. For the sites with no bar chart, in site D, the field of specimen time was populated with numbers, not in time format. In sites I and K, the field of specimen time was populated with a NULL value, and in site L, the field of specimen time was populated with zero in time format.

For assessing the ability of some rule templates to determine suboptimal clinical care quality, we used the drug and laboratory rule template to check for ordering PSA for patients who received TRT prescriptions. Although the American Urological Association and the Endocrine Society recommend PSA screening before starting testosterone therapy and during the treatment period for male patients over 40 years of age, previous studies showed many male patients were prescribed TRT without testing their PSA [22,23]. This could be due to the controversies between the different scientific societies and their guidelines [24,25]. Our finding showed the rule captured many discrepancies and clear variability among sites. Even though we used laboratory and procedure data to find the order for the screening test, the discrepancies are still significant in most sites. The reason for these discrepancies could be due to the test being done in different healthcare systems, data entry errors, the different practices of clinical care due to the lack of consensus among the scientific societies to order this test before or during the TRT period, or issues with the quality of clinical care. Therefore, seeking clinician feedback to refine, validate, or add clinical knowledge specifications based on real-world practice will strengthen rules to capture data quality errors and assist in diagnosing the underlying causes.

Our findings show many sites have data quality issues mainly due to the incompleteness of data. The big challenge for most sites was keeping complete laboratory and medication data, which revealed obvious variability in data quality among sites. The framework was efficient in capturing data errors and scalable to run in a reasonable time, and rules were easy to implement on different sites using SQL. Also, the rule templates in the framework were able to capture many discrepancies which are often challenging to detect during the data curation process. Another strength of this study is that it shows the rule templates' ability to detect data errors, which may help determine variability in clinical care or clinical care quality problems. The findings of our study should be interpreted with consideration of potential limitations. The rules were effective in data error detection; however, more analyses are still needed to identify and confirm the root causes of data error. Rules that used laboratory value ranges that are different from the value ranges determined by various institutions depending on their requirement for laboratory equipment may capture more discrepancies. In addition, these rules still need validation to rule out any false-positive results. Finally, although the rule templates could explore the data quality for many sites and capture the discrepancies in data, we need to come up with scores that categorize sites depending on their data quality check.

### Future direction

Given the framework’s efficiency in detecting data inconsistency, we seek to determine the concordance of EHR-CDM-based data and claim data in GROUSE. We are looking to create a scoring algorithm that will produce data quality scores depending on the results produced by implementing the framework. This will facilitate the interaction with clinicians during the research process or when we need feedback on the quality of clinical care concerns that appeared during the data quality assessment process. Another aspect on which to focus is developing a method to automate the implementation of this framework to make the data quality process faster and conducted on a regular basis.

## Conclusion

The large volume of healthcare data available requires adopting a comprehensive method to evaluate the quality of these data. In this study, we used rules tailored to run against the PCORnet CDM in previous work to check for data quality across multiple healthcare systems.

The framework showed strong performance, scalability, and ability to capture data errors. The rule templates showed promising results in detecting the possible quality of clinical care issues. The assessment revealed most sites had struggled with laboratory and medication data, and many sites have an issue with the completeness of data especially for laboratory units and specimen collection time. These rules capture many discrepancies due to the use of granular clinical information, which augments the current data curation process. More studies are needed to refine the rules using more knowledge sources and feedback from clinical experts as well as informatics teams that can integrate data quality improvement into clinical research networks and learning health systems.

**Table 1.** Summary for number of patients who received TRT and not tested for PSA

Clinical site	Count of patients who received TRT^a^ (n)	Count of patients who have no order of PSA^b^ (n)	The percentage of discrepancy^c^ (%)
Site A	352	118	33.5
Site B	959	129	13.5
Site C	1,149	137	11.9
Site D	174	31	17.8
Site E	79	31	39.2
Site F	3,406	1,449	42.5
Site G	6,396	1,108	17.3
Site H	3,221	1,613	50.1
Site I	165	84	50.9
Site J	260	169	65.0
Site K	2,587	643	24.9
Site L	1,268	788	62.1
Site M	390	42	10.8

a TRT; Testosterone Replacement Therapy.

b PSA; Prostate Specific Antigen.

c The percentage calculated by using the count of patients who have no PSA order as nominator and patients who received TRT as dominator.

## Supplementary material

For supplementary material accompanying this paper visit https://doi.org/10.1017/cts.2023.548. click here to view supplementary material
