# Supplementary material for: Electronic health record data quality variability across a multistate clinical research network
Source: J Clin Transl Sci. 2023 May 15;7(1):e130. doi: 10.1017/cts.2023.548 (PMC10308424; doi:10.1017/cts.2023.548)
Supplement: Supplementary file 1 [file S2059866123005484sup001.docx]

**Supplementary Materials**

**Table.1** Results Summary after Implementing Rules on All Sites (Patient level)

| Site A | | | | |
| --- | --- | --- | --- | --- |
| Template name | | Count of patients in the whole cohort (n) | Count of patients with discrepancies (n) | The percent of discrepancies (%) |
| *Out of range values* | | | | |
| Demographic data elements | | 2,117,840 | 0 | 0 |
| Observation data elements | | 822,871 | 13,015 | 1.6 |
| Valid laboratory values | | 399,628 | 48,663 | 12.2 |
| *Incompatibility* | | | | |
| Age and diagnosis | | 1,074,023 | 5,567 | 0.52 |
| Age and procedure | | 944,319 | 27,981 | 2.9 |
| Gender and diagnosis | | 151,690 | 6,372 | 4.2 |
| Gender and procedure | | 164,600 | 464 | 0.3 |
| Drug and diagnosis | | 1,321 | 61 | 4.6 |
| Drug and drug interaction | | 758,820 | 5,784 | 0.8 |
| Inpatient only procedure | | 879,823 | 13,004 | 1.5 |
| Diagnosis and laboratory | | 45,553 | 1,103 | 2.4 |
| *Incompleteness* | | | | |
| Drug and laboratory | | 54,245 | 10,046 | 18.5 |
| Drug and continuous procedure | | 5,337 | 234 | 4.4 |
| Drug monitoring | | 135,072 | 63,004 | 46.6 |
| *Date and time error* | | | | |
| Laboratory time | | 15,586 | 15,505 | 99.4 |
| Date in future | Death date | 238,490 | 0 | 0.0 |
|  | Birth date | 2,117,840 | 0 | 0.0 |
|  | Medicine administration date | 458,557 | 0 | 0.0 |
|  | Procedure date | 988,518 | 0 | 0.0 |
| *Duplication* | | | | |
| Duplication | | 988,518 | 0 | 0.0 |
| Site B | | | | |
| Template name | | Count of patients in the whole cohort (n) | Count of patients with discrepancies (n) | The percent of discrepancies (%) |
| *Out of range values* | | | | |
| Demographic data elements | | 1,088,260 | 0 | 0 |
| Observation data elements | | 1,169,141 | 1,782 | 0.16 |
| Valid laboratory values | | 743,275 | 381,046 | 51.3 |
| *Incompatibility* | | | | |
| Age and diagnosis | | 1,361,303 | 2,883 | 0.21 |
| Age and procedure | | 1,330,622 | 36,190 | 2.7 |
| Gender and diagnosis | | 279,329 | 5,633 | 2.0 |
| Gender and procedure | | 300,825 | 527 | 0.2 |
| Drug and diagnosis | | 998 | 54 | 5.4 |
| Drug and drug interaction | | 892,201 | 5,730 | 0.6 |
| Inpatient only procedure | | 960,125 | 3,032 | 0.3 |
| Diagnosis and laboratory | | 73,809 | 2,354 | 3.2 |
| *Incompleteness* | | | | |
| Drug and laboratory | | 115,850 | 10,134 | 8.7 |
| Drug and continuous procedure | | 6,299 | 181 | 2.9 |
| Drug monitoring | | 102,342 | 35,674 | 34.9 |
| *Date and time error* | | | | |
| Laboratory time | | 41,364 | 29,087 | 70.3 |
| Date in future | Death date | 106,308 | 0 | 0.0 |
|  | Birth date | 1,49,417 | 0 | 0.0 |
|  | Medicine administration date | 600,693 | 0 | 0.0 |
|  | Procedure date | 1,330,643 | 3 | 0.0002 |
| *Duplication* | | | | |
| Duplication | | 1,330,643 | 0 | 0.0 |
| Site C | | | | |
| Template name | | Count of patients in the whole cohort (n) | Count of patients with discrepancies (n) | The percent of discrepancies (%) |
| *Out of range values* | | | | |
| Demographic data elements | | 1,088,260 | 0 | 0 |
| Observation data elements | | 812,087 | 25,952 | 2.4 |
| Valid laboratory values | | 651,388 | 487,934 | 74.9 |
| *Incompatibility* | | | | |
| Age and diagnosis | | 1,037,662 | 6,228 | 0.6 |
| Age and procedure | | 883,571 | 75,978 | 8.6 |
| Gender and diagnosis | | 238,245 | 1,684 | 0.7 |
| Gender and procedure | | 242,975 | 39 | 0.02 |
| Drug and diagnosis | | 710 | 8 | 1.1 |
| Drug and drug interaction | | 805,970 | 5,075 | 0.6 |
| Inpatient only procedure | | 933,800 | 14,400 | 1.5 |
| Diagnosis and laboratory | | 58,635 | 498 | 0.9 |
| *Incompleteness* | | | | |
| Drug and laboratory | | 149,129 | 14,870 | 10.0 |
| Drug and continuous procedure | | 6,852 | 206 | 3.0 |
| Drug monitoring | | 76,135 | 21,448 | 28.2 |
| *Date and time error* | | | | |
| Laboratory time | | 29,707 | 16,938 | 57.0 |
| Date in future | Death date | 84,126 | 0 | 0.0 |
|  | Birth date | 1,088,260 | 0 | 0.0 |
|  | Medicine administration date | 241,481 | 0 | 0.0 |
|  | Procedure date | 954,479 | 0 | 0.0 |
| *Duplication* | | | | |
| Duplication | | 954,479 | 0 | 0.0 |
| Site D | | | | |
| Template name | | Count of patients in the whole cohort (n) | Count of patients with discrepancies (n) | The percent of discrepancies (%) |
| *Out of range values* | | | | |
| Demographic data elements | | 441,409 | 0 | 0 |
| Observation data elements | | 440,307 | 8,636 | 1.9 |
| Valid laboratory values | | 305,094 | 253,895 | 83.2 |
| *Incompatibility* | | | | |
| Age and diagnosis | | 414,060 | 1,559 | 0.38 |
| Age and procedure | | 415,140 | 2,827 | 0.7 |
| Gender and diagnosis | | 97,584 | 1,329 | 1.4 |
| Gender and procedure | | 47,152 | 45 | 0.1 |
| Drug and diagnosis | | 258 | 2 | 0.8 |
| Drug and drug interaction | | 341,748 | 708 | 0.2 |
| Inpatient only procedure | | 364,971 | 40 | 0.01 |
| Diagnosis and laboratory | | 48,770 | 982 | 2.0 |
| *Incompleteness* | | | | |
| Drug and laboratory | | 35,933 | 1,453 | 4.0 |
| Drug and continuous procedure | | 1,566 | 63 | 4.0 |
| Drug monitoring | | 21,868 | 3,018 | 13.8 |
| *Date and time error* | | | | |
| Laboratory time | | 4,294 | 4,262 | 99.3 |
| Date in future | Death date | 26,149 | 0 | 0.0 |
|  | Birth date | 441,409 | 0 | 0.0 |
|  | Medicine administration date | 145,065 | 0 | 0.0 |
|  | Procedure date | 417,777 | 0 | 0.0 |
| *Duplication* | | | | |
| Duplication | | 417,777 | 0 | 0.0 |
| Site E | | | | |
| Template name | | Count of patients in the whole cohort (n) | Count of patients with discrepancies (n) | The percent of discrepancies (%) |
| *Out of range values* | | | | |
| Demographic data elements | | 1,615,741 | 0 | 0 |
| Observation data elements | | 788,881 | 5,744 | 0.73 |
| Valid laboratory values | | 633,149 | 430,412 | 67.9 |
| *Incompatibility* | | | | |
| Age and diagnosis | | 750,607 | 3,820 | 0.51 |
| Age and procedure | | 775,732 | 8,421 | 1.1 |
| Gender and diagnosis | | 116,179 | 2,551 | 2.2 |
| Gender and procedure | | 152,107 | 440 | 0.3 |
| Drug and diagnosis | | 239 | 4 | 1.7 |
| Drug and drug interaction | | 680,157 | 137 | 0.02 |
| Inpatient only procedure | | 563,272 | 707 | 0.1 |
| Diagnosis and laboratory | | 34,857 | 1,519 | 4.3 |
| *Incompleteness* | | | | |
| Drug and laboratory | | 1,115 | 43 | 3.9 |
| Drug and continuous procedure | | 106 | 1 | 0.9 |
| Drug monitoring | | 61,392 | 21,776 | 35.5 |
| *Date and time error* | | | | |
| Laboratory time | | 25,644 | 18,368 | 71.6 |
| Date in future | Death date | 267,617 | 0 | 0.0 |
|  | Birth date | 1,615,784 | 0 | 0.0 |
|  | Medicine administration date | 436,248 | 0 | 0.0 |
|  | Procedure date | 775,747 | 0 | 0.0 |
| *Duplication* | | | | |
| Duplication | | 775,747 | 0 | 0.0 |
| Site F | | | | |
| Template name | | Count of patients in the whole cohort (n) | Count of patients with discrepancies (n) | The percent of discrepancies (%) |
| *Out of range values* | | | | |
| Demographic data elements | | 2,511,983 | 0 | 0 |
| Observation data elements | | 2,267,288 | 7,083 | 0.31 |
| Valid laboratory values | | 1,427,763 | 1,038,927 | 72.8 |
| *Incompatibility* | | | | |
| Age and diagnosis | | 2,434,480 | 8,922 | 0.37 |
| Age and procedure | | 2,315,985 | 108,073 | 4.7 |
| Gender and diagnosis | | 671,807 | 3,219 | 0.5 |
| Gender and procedure | | 594,880 | 227 | 0.1 |
| Drug and diagnosis | | 552 | 12 | 2.2 |
| Drug and drug interaction | | 2,144,475 | 392 | 0.02 |
| Inpatient only procedure | | 2,098,510 | 6,243 | 0.3 |
| Diagnosis and laboratory | | 160,795 | 3,949 | 2.5 |
| *Incompleteness* | | | | |
| Drug and laboratory | | 235,548 | 43,069 | 18.3 |
| Drug and continuous procedure | | 10,971 | 920 | 8.4 |
| Drug monitoring | | 99,720 | 37,730 | 37.8 |
| *Date and time error* | | | | |
| Laboratory time | | 28,758 | 19,663 | 68.4 |
| Date in future | Death date | 141,014 | 0 | 0.0 |
|  | Birth date | 2,511,597 | 0 | 0.0 |
|  | Medicine administration date | 1,356,178 | 0 | 0.0 |
|  | Procedure date | 2,355,861 | 1 | 0.00004 |
| *Duplication* | | | | |
| Duplication | | 2,355,861 | 0 | 0.0 |
| Site G | | | | |
| Template name | | Count of patients in the whole cohort (n) | Count of patients with discrepancies (n) | The percent of discrepancies (%) |
| *Out of range values* | | | | |
| Demographic data elements | | 4,088,171 | 0 | 0 |
| Observation data elements | | 3,628,875 | 31,550 | 0.87 |
| Valid laboratory values | | 2,149,766 | 569,581 | 26.5 |
| *Incompatibility* | | | | |
| Age and diagnosis | | 4,087,962 | 15,941 | 0.39 |
| Age and procedure | | 4,040,261 | 193,427 | 4.8 |
| Gender and diagnosis | | 840,910 | 7,002 | 0.8 |
| Gender and procedure | | 767,060 | 708 | 0.1 |
| Drug and diagnosis | | 5,166 | 275 | 5.3 |
| Drug and drug interaction | | 3,458,698 | 8,579 | 0.2 |
| Inpatient only procedure | | 3,607,084 | 47,496 | 1.3 |
| Diagnosis and laboratory | | 156,552 | 4,400 | 2.8 |
| *Incompleteness* | | | | |
| Drug and laboratory | | 381,484 | 52,541 | 13.8 |
| Drug and continuous procedure | | 8,932 | 340 | 3.8 |
| Drug monitoring | | 202,455 | NA^*^ | NA |
| *Date and time error* | | | | |
| Laboratory time | |  |  |  |
| Date in future | Death date | 161,921 | 0 | 0.0 |
|  | Birth date | 4,088,171 | 0 | 0.0 |
|  | Medicine administration date | NA | NA | NA |
|  | Procedure date | 4,040,278 | 0 | 0.0 |
| *Duplication* | | | | |
| Duplication | | 4,040,278 | 0 | 0.0 |
| Site H | | | | |
| Template name | | Count of patients in the whole cohort (n) | Count of patients with discrepancies (n) | The percent of discrepancies (%) |
| *Out of range values* | | | | |
| Demographic data elements | | 2,935,436 | 0 | 0 |
| Observation data elements | | 916,887 | 8,264 | 0.9 |
| Valid laboratory values | | 516,961 | 391,529 | 75.7 |
| *Incompatibility* | | | | |
| Age and diagnosis | | 1,082,734 | 2,570 | 0.24 |
| Age and procedure | | 1,004,151 | 27,010 | 2.7 |
| Gender and diagnosis | | 274,771 | 9,555 | 3.5 |
| Gender and procedure | | 166,702 | 374 | 0.2 |
| Drug and diagnosis | | 1,672 | 148 | 8.9 |
| Drug and drug interaction | | 897,394 | 12,647 | 1.4 |
| Inpatient only procedure | | 861,649 | 37,444 | 4.3 |
| Diagnosis and laboratory | | 77,869 | 5,655 | 7.3 |
| *Incompleteness* | | | | |
| Drug and laboratory | | 79,508 | 6,536 | 8.2 |
| Drug and continuous procedure | | 10,723 | 659 | 6.1 |
| Drug monitoring | | 106,296 | 43,184 | 40.6 |
| *Date and time error* | | | | |
| Laboratory time | | 23,277 | 16,574 | 71.2 |
| Date in future | Death date | 312,714 | 6,112 | 2.0 |
|  | Birth date | 2,935,436 | 0 | 0.0 |
|  | Medicine administration date | 536,939 | 0 | 0.0 |
|  | Procedure date | 1,004,337 | 7 | 0.001 |
| *Duplication* | | | | |
| Duplication | | 1,004,337 | 0 | 0.0 |
| Site I | | | | |
| Template name | | Count of patients in the whole cohort (n) | Count of patients with discrepancies (n) | The percent of discrepancies (%) |
| *Out of range values* | | | | |
| Demographic data elements | | 2,406,425 | 0 | 0 |
| Observation data elements | | 924,615 | 4,592 | 0.5 |
| Valid laboratory values | | 330,882 | 328,948 | 99.4 |
| *Incompatibility* | | | | |
| Age and diagnosis | | 1,991,103 | 2,467 | 0.12 |
| Age and procedure | | 1,993,283 | 43,090 | 2.2 |
| Gender and diagnosis | | 304,063 | 1,423 | 0.5 |
| Gender and procedure | | 237,138 | 304 | 0.1 |
| Drug and diagnosis | | 130 | 0 | 0 |
| Drug and drug interaction | | 883,661 | 559 | 0.1 |
| Inpatient only procedure | | 1,778,216 | 8,813 | 0.5 |
| Diagnosis and laboratory | | 38,448 | 16,707 | 43.5 |
| *Incompleteness* | | | | |
| Drug and laboratory | | 27,445 | 22,957 | 83.6 |
| Drug and continuous procedure | | 6,653 | 171 | 2.6 |
| Drug monitoring | | 21,868 | 3,018 | 13.8 |
| *Date and time error* | | | | |
| Laboratory time | | 11,883 | NA | NA |
| Date in future | Death date | 17,396 | 0 | 0.0 |
|  | Birth date | 2,206,452 | 0 | 0.0 |
|  | Medicine administration date | 20,647 | 0 | 0.0 |
|  | Procedure date | 1,993,283 | 0 | 0.0 |
| *Duplication* | | | | |
| Duplication | | 1,993,283 | 0 | 0.0 |
| Site J | | | | |
| Template name | | Count of patients in the whole cohort (n) | Count of patients with discrepancies (n) | The percent of discrepancies (%) |
| *Out of range values* | | | | |
| Demographic data elements | | 4,491,223 | 0 | 0 |
| Observation data elements | | 874,505 | 5,215 | 0.6 |
| Valid laboratory values | | 513,395 | 380,817 | 74.2 |
| *Incompatibility* | | | | |
| Age and diagnosis | | 3,133,489 | 10,809 | 0.35 |
| Age and procedure | | 2,825,371 | 16,027 | 0.6 |
| Gender and diagnosis | | 580,343 | 36,643 | 6.3 |
| Gender and procedure | | 594,619 | 1,208 | 0.2 |
| Drug and diagnosis | | 705 | 19 | 2.7 |
| Drug and drug interaction | | 857,425 | 8,071 | 1.0 |
| Inpatient only procedure | | 1,732,974 | 33,373 | 1.9 |
| Diagnosis and laboratory | | 64,469 | 5,764 | 8.9 |
| *Incompleteness* | | | | |
| Drug and laboratory | | 64,830 | 8,480 | 13.0 |
| Drug and continuous procedure | | 9,260 | 393 | 4.2 |
| Drug monitoring | | 125,387 | 46,936 | 37.4 |
| *Date and time error* | | | | |
| Laboratory time | | 29,355 | 21,355 | 72.7 |
| Date in future | Death date | 38,835 | 0 | 0.0 |
|  | Birth date | 4,491,223 | 6 | 0.0001 |
|  | Medicine administration date | 430,914 | 0 | 0.0 |
|  | Procedure date | 2,826,222 | 0 | 0.0 |
| *Duplication* | | | | |
| Duplication | | 2,826,222 | 0 | 0.0 |
| Site K | | | | |
| Template name | | Count of patients in the whole cohort (n) | Count of patients with discrepancies (n) | The percent of discrepancies (%) |
| *Out of range values* | | | | |
| Demographic data elements | | 1,382,487 | 0 | 0 |
| Observation data elements | | 1,184,710 | 2,078 | 0.18 |
| Valid laboratory values | | 589,710 | 270,905 | 45.9 |
| *Incompatibility* | | | | |
| Age and diagnosis | | 1,341,806 | 2,794 | 0.21 |
| Age and procedure | | 1,260,316 | 41,507 | 3.3 |
| Gender and diagnosis | | 251,645 | 2,960 | 1.2 |
| Gender and procedure | | 250,481 | 697 | 0.3 |
| Drug and diagnosis | | 1,211 | 80 | 6.6 |
| Drug and drug interaction | | 943,395 | 4,316 | 0.5 |
| Inpatient only procedure | | 1,171,884 | 5,824 | 0.5 |
| Diagnosis and laboratory | | 63,981 | 3,545 | 5.5 |
| *Incompleteness* | | | | |
| Drug and laboratory | | 67,582 | 7,909 | 11.7 |
| Drug and continuous procedure | | 6,882 | 128 | 1.9 |
| Drug monitoring | | 96,051 | 44,115 | 45.9 |
| *Date and time error* | | | | |
| Laboratory time | | 38,546 | NA | NA |
| Date in future | Death date | 32,732 | 0 | 0.0 |
|  | Birth date | 1,382,487 | 0 | 0.0 |
|  | Medicine administration date | 497,991 | 0 | 0.0 |
|  | Procedure date | 1,260,447 | 0 | 0.0 |
| *Duplication* | | | | |
| Duplication | | 1,260,447 | 0 | 0.0 |
| Site L | | | | |
| Template name | | Count of patients in the whole cohort (n) | Count of patients with discrepancies (n) | The percent of discrepancies (%) |
| *Out of range values* | | | | |
| Demographic data elements | | 5,034,747 | 59 | 0.003 |
| Observation data elements | | 2,695,192 | 37,065 | 1.4 |
| Valid laboratory values | | 1,694,190 | 1,448,076 | 85.5 |
| *Incompatibility* | | | | |
| Age and diagnosis | | 1,405,399 | 2,914 | 0.21 |
| Age and procedure | | 2,220,484 | 31,648 | 1.4 |
| Gender and diagnosis | | 223,544 | 1,574 | 0.7 |
| Gender and procedure | | 206,475 | 175 | 0.1 |
| Drug and diagnosis | | 705 | 15 | 2.1 |
| Drug and drug interaction | | 2,376,555 | 22,956 | 1.0 |
| Inpatient only procedure | | 2,134,068 | 79,359 | 3.7 |
| Diagnosis and laboratory | | 57,068 | 2,075 | 3.6 |
| *Incompleteness* | | | | |
| Drug and laboratory | | 160,482 | 10,236 | 6.4 |
| Drug and continuous procedure | | 10,875 | 3,543 | 32.6 |
| Drug monitoring | | 377,246 | 177,486 | 47.0 |
| *Date and time error* | | | | |
| Laboratory time | | 50,343 | NA | NA |
| Date in future | Death date | 169,948 | 0 | 0.0 |
|  | Birth date | 5,034,747 | 0 | 0.0 |
|  | Medicine administration date | NA | NA | NA |
|  | Procedure date | 2,458,718 | 0 | 0.0 |
| *Duplication* | | | | |
| Duplication | | 2,458,718 | 0 | 0.0 |
| Site M | | | | |
| Template name | | Count of patients in the whole cohort (n) | Count of patients with discrepancies (n) | The percent of discrepancies (%) |
| *Out of range values* | | | | |
| Demographic data elements | | 1,258,443 | 0 | 0 |
| Observation data elements | | 999,721 | 632 | 0.1 |
| Valid laboratory values | | 176,904 | 64,193 | 36.3 |
| *Incompatibility* | | | | |
| Age and diagnosis | | 1,133,083 | 3,934 | 0.35 |
| Age and procedure | | 1,072,824 | 26,718 | 2.5 |
| Gender and diagnosis | | 252,353 | 10,220 | 4.0 |
| Gender and procedure | | 194,248 | 535 | 0.3 |
| Drug and diagnosis | | 536 | 8 | 1.5 |
| Drug and drug interaction | | 722,673 | 1,332 | 0.2 |
| Inpatient only procedure | | 1,034,994 | 7,845 | 0.8 |
| Diagnosis and laboratory | | 24,290 | 3,923 | 16.2 |
| *Incompleteness* | | | | |
| Drug and laboratory | | 30,270 | 9,091 | 30.0 |
| Drug and continuous procedure | | 6,310 | 76 | 1.2 |
| Drug monitoring | | 43,313 | 4,812 | 11.1 |
| *Date and time error* | | | | |
| Laboratory time | | 4,762 | 3,410 | 71.6 |
| Date in future | Death date | 66,398 | 0 | 0.0 |
|  | Birth date | 1,258,443 | 0 | 0.0 |
|  | Medicine administration date | 646,920 | 0 | 0 |
|  | Procedure date | 1,094,926 | 3 | 0.0003 |
| *Duplication* | | | | |
| Duplication | | 1,094,926 | 0 | 0.0 |

^*^NA; data element needed to run the rule is not available in the database.

**Table. 2** Results Summary after Implementing Rules on All Sites (Encounter level)

| Site A | |  |  |  |
| --- | --- | --- | --- | --- |
| Template name | | Count of observations in the whole cohort (n) | Count of observations with discrepancies (n) | The percent of discrepancies (%) |
| *Out of range values* | | | | |
| Demographic data elements | | 2,118,936 | 0 | 0 |
| Observation data elements | | 34,161,712 | 21,093 | 0.1 |
| Valid laboratory values | | 23,998,535 | 57,895 | 0.2 |
| *Incompatibility* | | | | |
| Age and diagnosis | | 60,182,380 | 11,787 | 0.02 |
| Age and procedure | | 39,453,903 | 49,366 | 0.1 |
| Gender and diagnosis | | 3,182,490 | 11,355 | 0.4 |
| Gender and procedure | | 891,169 | 464 | 0.1 |
| Drug and diagnosis | | 18,113 | 79 | 0.4 |
| Drug and drug interaction | | 118,481,570 | 44,319 | 0.04 |
| Inpatient only procedure | | 29,424,255 | 14,756 | 0.1 |
| Diagnosis and laboratory | | 114,301,061 | 107,051 | 0.1 |
| *Incompleteness* | | | | |
| Drug and laboratory | | 18,755,780 | 238,289 | 1.3 |
| Drug and continuous procedure | | 6,015,499 | 70,289 | 1.2 |
| Drug monitoring | | 28,760,053 | 10,313,643 | 35.9 |
| *Date and time error* | | | | |
| Laboratory time | | 48,216 | 47,730 | 98.9 |
| Date in future | Death date | 282,155 | 0 | 0.0 |
|  | Birth date | 2,118,936 | 0 | 0.0 |
|  | Medicine administration date | 36,142,612 | 0 | 0.0 |
|  | Procedure date | 43,969,350 | 0 | 0.0 |
| *Duplication* | | | | |
| Duplication | | 43,969,350 | 0 | 0.0 |
| Site B | |  |  |  |
| Template name | | Count of observations in the whole cohort (n) | Count of observations with discrepancies (n) | The percent of discrepancies (%) |
| *Out of range values* | | | | |
| Demographic data elements | | 1,490,511 | 0 | 0 |
| Observation data elements | | 59,274,368 | 1,849 | 0.003 |
| Valid laboratory values | | 65,772,492 | 626,597 | 0.95 |
| *Incompatibility* | | | | |
| Age and diagnosis | | 245,468,548 | 7,497 | 0.003 |
| Age and procedure | | 39,453,903 | 49,366 | 0.1 |
| Gender and diagnosis | | 12,730,890 | 22,851 | 0.2 |
| Gender and procedure | | 2,843,183 | 652 | 0.02 |
| Drug and diagnosis | | 16,024 | 96 | 0.6 |
| Drug and drug interaction | | 60,337,033 | 13,629 | 0.02 |
| Inpatient only procedure | | 91,272,263 | 3,424 | 0.004 |
| Diagnosis and laboratory | | 451,656,441 | 220,451 | 0.1 |
| *Incompleteness* | | | | |
| Drug and laboratory | | 60,045,231 | 208,225 | 0.3 |
| Drug and continuous procedure | | 13,444,893 | 98,454 | 0.7 |
| Drug monitoring | | 8,845,949 | 2,143,134 | 24.2 |
| *Date and time error* | | | | |
| Laboratory time | | 125,342 | 70,621 | 56.3 |
| Date in future | Death date | 106,347 | 0 | 0.0 |
|  | Birth date | 1,490,511 | 0 | 0.0 |
|  | Medicine administration date | 67,718,698 | 0 | 0.0 |
|  | Procedure date | 220,059,114 | 3 | 0.000001 |
| *Duplication* | | | | |
| Duplication | | 220,059,114 | 0 | 0.0 |
| Site C | |  |  |  |
| Template name | | Count of observations in the whole cohort (n) | Count of observations with discrepancies (n) | The percent of discrepancies (%) |
| *Out of range values* | | | | |
| Demographic data elements | | 1,331,394 | 0 | 0 |
| Observation data elements | | 27,063,109 | 47,536 | 0.2 |
| Valid laboratory values | | 89,159,668 | 1,657,483 | 1.9 |
| *Incompatibility* | | | | |
| Age and diagnosis | | 348,358,006 | 26,645 | 0.01 |
| Age and procedure | | 39,453,903 | 49,366 | 0.1 |
| Gender and diagnosis | | 16,553,108 | 9,734 | 0.1 |
| Gender and procedure | | 5,574,678 | 65 | 0.001 |
| Drug and diagnosis | | 42,766 | 53 | 0.1 |
| Drug and drug interaction | | 99,253,031 | 36,030 | 0.04 |
| Inpatient only procedure | | 105,825,782 | 17,983 | 0.02 |
| Diagnosis and laboratory | | 971,149,590 | 89,901 | 0.01 |
| *Incompleteness* | | | | |
| Drug and laboratory | | 82,213,584 | 457,884 | 0.6 |
| Drug and continuous procedure | | 23,786,736 | 15,743 | 0.1 |
| Drug monitoring | | 1,407,815 | 698,225 | 49.6 |
| *Date and time error* | | | | |
| Laboratory time | | 141,207 | 55,618 | 39.4 |
| Date in future | Death date | 92,973 | 0 | 0.0 |
|  | Birth date | 1,331,394 | 0 | 0.0 |
|  | Medicine administration date | 12,084,782 | 0 | 0.0 |
|  | Procedure date | 112,888,990 | 0 | 0.0 |
| *Duplication* | | | | |
| Duplication | | 112,888,990 | 0 | 0.0 |
| Site D | |  |  |  |
| Template name | | Count of observations in the whole cohort (n) | Count of observations with discrepancies (n) | The percent of discrepancies (%) |
| *Out of range values* | | | | |
| Demographic data elements | | 446,469 | 0 | 0 |
| Observation data elements | | 32,646,668 | 24,167 | 0.1 |
| Valid laboratory values | | 32,439,720 | 2,124,451 | 6.6 |
| *Incompatibility* | | | | |
| Age and diagnosis | | 27,947,414 | 2,691 | 0.01 |
| Age and procedure | | 39,453,903 | 49,366 | 0.1 |
| Gender and diagnosis | | 1,614,411 | 4,457 | 0.3 |
| Gender and procedure | | 111,904 | 48 | 0.04 |
| Drug and diagnosis | | 1,147 | 3 | 0.3 |
| Drug and drug interaction | | 10,380,005 | 1,231 | 0.01 |
| Inpatient only procedure | | 14,347,741 | 42 | 0.0003 |
| Diagnosis and laboratory | | 148,171,891 | 304,323 | 0.2 |
| *Incompleteness* | | | | |
| Drug and laboratory | | 10,935,586 | 31,469 | 0.3 |
| Drug and continuous procedure | | 542,873 | 5,892 | 1.1 |
| Drug monitoring | | 106,988 | 113,112 | 10.2 |
| *Date and time error* | | | | |
| Laboratory time | | 8,774 | 8,623 | 98.3 |
| Date in future | Death date | 26,264 | 0 | 0.0 |
|  | Birth date | 441,469 | 0 | 0.0 |
|  | Medicine administration date | 21,602,269 | 0 | 0.0 |
|  | Procedure date | 27,779,886 | 0 | 0.0 |
| *Duplication* | | | | |
| Duplication | | 27,779,886 | 0 | 0.0 |
| Site E | |  |  |  |
| Template name | | Count of observations in the whole cohort (n) | Count of observations with discrepancies (n) | The percent of discrepancies (%) |
| *Out of range values* | | | | |
| Demographic data elements | | 1,615,748 | 0 | 0 |
| Observation data elements | | 40,818,237 | 20,244 | 0.1 |
| Valid laboratory values | | 62,387,065 | 2,174,484 | 3.5 |
| *Incompatibility* | | | | |
| Age and diagnosis | | 28,764,674 | 64,855 | 0.23 |
| Age and procedure | | 39,453,903 | 49,366 | 0.13 |
| Gender and diagnosis | | 1,551,327 | 6,098 | 0.4 |
| Gender and procedure | | 611,879 | 609 | 0.1 |
| Drug and diagnosis | | 1,879 | 4 | 0.2 |
| Drug and drug interaction | | 47,688,847 | 231 | 0.0005 |
| Inpatient only procedure | | 13,890,133 | 808 | 0.01 |
| Diagnosis and laboratory | | 30,447,775 | 180,080 | 0.6 |
| *Incompleteness* | | | | |
| Drug and laboratory | | 178,769 | 713 | 0.4 |
| Drug and continuous procedure | | 18,885 | 5 | 0.03 |
| Drug monitoring | | 12,358,366 | 5,305,861 | 42.9 |
| *Date and time error* | | | | |
| Laboratory time | | 74,296 | 44,397 | 59.8 |
| Date in future | Death date | 267,619 | 0 | 0.0 |
|  | Birth date | 1,615,784 | 0 | 0.0 |
|  | Medicine administration date | 59,157,605 | 0 | 0.0 |
|  | Procedure date | 48,994,998 | 0 | 0.0 |
| *Duplication* | | | | |
| Duplication | | 48,994,998 | 0 | 0.0 |
| Site F | |  |  |  |
| Template name | | Count of observations in the whole cohort (n) | Count of observations with discrepancies (n) | The percent of discrepancies (%) |
| *Out of range values* | | | | |
| Demographic data elements | | 2,545,338 | 0 | 0 |
| Observation data elements | | 136,129,030 | 7,757 | 0.01 |
| Valid laboratory values | | 64,009,327 | 4,194,066 | 6.6 |
| *Incompatibility* | | | | |
| Age and diagnosis | | 280,431,066 | 23,264 | 0.01 |
| Age and procedure | | 39,453,903 | 49,366 | 0.13 |
| Gender and diagnosis | | 15,590,737 | 9,298 | 0.1 |
| Gender and procedure | | 4,155,548 | 341 | 0.01 |
| Drug and diagnosis | | 7,316 | 12 | 0.2 |
| Drug and drug interaction | | 147,842,756 | 832 | 0.01 |
| Inpatient only procedure | | 169,833,666 | 6,870 | 0.0005 |
| Diagnosis and laboratory | | 1,025,677,280 | 525,293 | 0.5 |
| *Incompleteness* | | | | |
| Drug and laboratory | | 41,760,525 | 1,108,056 | 2.7 |
| Drug and continuous procedure | | 9,946,431 | 165,722 | 1.7 |
| Drug monitoring | | 3,246,387 | 1,781,425 | 54.9 |
| *Date and time error* | | | | |
| Laboratory time | | 45,990 | 28,492 | 62.0 |
| Date in future | Death date | 141,014 | 0 | 0.0 |
|  | Birth date | 2,511,597 | 0 | 0.0 |
|  | Medicine administration date | 124,807,460 | 0 | 0.0 |
|  | Procedure date | 240,564,578 | 3 | 0.00000001 |
| *Duplication* | | | | |
| Duplication | | 240,546,578 | 0 | 0.0 |
| Site G | |  |  |  |
| Template name | | Count of observations in the whole cohort (n) | Count of observations with discrepancies (n) | The percent of discrepancies (%) |
| *Out of range values* | | | | |
| Demographic data elements | | 4,088,175 | 0 | 0 |
| Observation data elements | | 103,146,771 | 50,442 | 0.1 |
| Valid laboratory values | | 107,396,061 | 756,763 | 0.7 |
| *Incompatibility* | | | | |
| Age and diagnosis | | 178,862,310 | 24,364 | 0.01 |
| Age and procedure | | 39,453,903 | 49,366 | 0.13 |
| Gender and diagnosis | | 10,236,807 | 14,417 | 0.1 |
| Gender and procedure | | 3,459,742 | 902 | 0.03 |
| Drug and diagnosis | | 22,215 | 284 | 1.3 |
| Drug and drug interaction | | 127,787,116 | 17,418 | 0.01 |
| Inpatient only procedure | | 151,510,356 | 61,013 | 0.04 |
| Diagnosis and laboratory | | 41,934,977 | 25,750,592 | 61.4 |
| *Incompleteness* | | | | |
| Drug and laboratory | | 26,456,070 | 394,815 | 1.5 |
| Drug and continuous procedure | | 3,786,895 | 85,444 | 2.3 |
| Drug monitoring | | 5,350,241 | NA^*^ | NA |
| *Date and time error* | | | | |
| Laboratory time | | NA | NA | NA |
| Date in future | Death date | 161,921 | 0 | 0.0 |
|  | Birth date | 4,088,171 | 0 | 0.0 |
|  | Medicine administration date | NA | NA | NA |
|  | Procedure date | 275,416,010 | 0 | 0.0 |
| *Duplication* | | | | |
| Duplication | | 275,416,010 | 0 | 0.0 |
| Site H | |  |  |  |
| Template name | | Count of observations in the whole cohort (n) | Count of observations with discrepancies (n) | The percent of discrepancies (%) |
| *Out of range values* | | | | |
| Demographic data elements | | 2,935,461 | 0 | 0 |
| Observation data elements | | 51,418,983 | 18,362 | 0.04 |
| Valid laboratory values | | 117,545,061 | 2,402,230 | 2.0 |
| *Incompatibility* | | | | |
| Age and diagnosis | | 107,320,034 | 9,463 | 0.01 |
| Age and procedure | | 39,453,903 | 49,366 | 0.13 |
| Gender and diagnosis | | 5,597,832 | 25,883 | 0.5 |
| Gender and procedure | | 628,640 | 794 | 0.1 |
| Drug and diagnosis | | 10,454 | 199 | 1.9 |
| Drug and drug interaction | | 62,982,853 | 34,207 | 0.05 |
| Inpatient only procedure | | 34,317,945 | 48,790 | 0.1 |
| Diagnosis and laboratory | | 141,758,007 | 459,229 | 0.3 |
| *Incompleteness* | | | | |
| Drug and laboratory | | 30,003,047 | 83,577 | 0.3 |
| Drug and continuous procedure | | 6,019,508 | 98,326 | 1.6 |
| Drug monitoring | | 5,802,805 | 2,099,658 | 36.2 |
| *Date and time error* | | | | |
| Laboratory time | | 125,239 | 37,362 | 29.8 |
| Date in future | Death date | 392,335 | 6,112 | 1.6 |
|  | Birth date | 2,935,461 | 0 | 0.0 |
|  | Medicine administration date | 94,244,826 | 0 | 0.0 |
|  | Procedure date | 70,637,283 | 7 | 0.00001 |
| *Duplication* | | | | |
| Duplication | | 70,637,283 | 0 | 0.0 |
| Site I | |  |  |  |
| Template name | | Count of observations in the whole cohort (n) | Count of observations with discrepancies (n) | The percent of discrepancies (%) |
| *Out of range values* | | | | |
| Demographic data elements | | 2,406,452 | 0 | 0 |
| Observation data elements | | 26,980.811 | 4,909 | 0.02 |
| Valid laboratory values | | 6,501,395 | 4,090,536 | 62.9 |
| *Incompatibility* | | | | |
| Age and diagnosis | | 68,778,109 | 6,370 | 0.01 |
| Age and procedure | | 39,453,903 | 49,366 | 0.13 |
| Gender and diagnosis | | 3,707,710 | 3,929 | 0.1 |
| Gender and procedure | | 1,046,393 | 517 | 0.1 |
| Drug and diagnosis | | 485 | 0 | 0 |
| Drug and drug interaction | | 9,114,549 | 583 | 0.01 |
| Inpatient only procedure | | 25,451,729 | 9,557 | 0.04 |
| Diagnosis and laboratory | | 12,248,806 | 2,837,761 | 23.2 |
| *Incompleteness* | | | | |
| Drug and laboratory | | 5,037,406 | 560,514 | 11.1 |
| Drug and continuous procedure | | 118,273 | 720 | 0.6 |
| Drug monitoring | | 1,106,988 | 113,112 | 10.2 |
| *Date and time error* | | | | |
| Laboratory time | | 24,056 | NA | NA |
| Date in future | Death date | 17,396 | 0 | 0.0 |
|  | Birth date | 2,406,452 | 0 | 0.0 |
|  | Medicine administration date | 264,536 | 0 | 0.0 |
|  | Procedure date | 39,543,475 | 0 | 0.0 |
| *Duplication* | | | | |
| Duplication | | 1,993,283 | 0 | 0.0 |
| Site J | |  |  |  |
| Template name | | Count of observations in the whole cohort (n) | Count of observations with discrepancies (n) | The percent of discrepancies (%) |
| *Out of range values* | | | | |
| Demographic data elements | | 4,491,864 | 0 | 0 |
| Observation data elements | | 7,784,469 | 12,171 | 0.2 |
| Valid laboratory values | | 37,725,518 | 1,017,120 | 2.7 |
| *Incompatibility* | | | | |
| Age and diagnosis | | 177,999,351 | 31,630 | 0.02 |
| Age and procedure | | 39,453,903 | 49,366 | 0.13 |
| Gender and diagnosis | | 14,086,977 | 98,439 | 0.7 |
| Gender and procedure | | 2,565,311 | 1,300 | 0.1 |
| Drug and diagnosis | | 7000 | 21 | 0.003 |
| Drug and drug interaction | | 51,895,033 | 20,774 | 0.04 |
| Inpatient only procedure | | 60,747,053 | 40,129 | 0.1 |
| Diagnosis and laboratory | | 255,316,651 | 953,010 | 0.4 |
| *Incompleteness* | | | | |
| Drug and laboratory | | 44,820,827 | 200,459 | 0.4 |
| Drug and continuous procedure | | 5,098,424 | 62,431 | 1.2 |
| Drug monitoring | | 13,566,487 | 3,069,341 | 22.6 |
| *Date and time error* | | | | |
| Laboratory time | | 99,746 | 61,331 | 62.0 |
| Date in future | Death date | 38,835 | 0 | 0.0 |
|  | Birth date | 4,491,223 | 6 | 0.0001 |
|  | Medicine administration date | 58,890,802 | 0 | 0.0 |
|  | Procedure date | 151,220,597 | 0 | 0.0 |
| *Duplication* | | | | |
| Duplication | | 151,220,597 | 0 | 0.0 |
| Site K | |  |  |  |
| Template name | | Count of observations in the whole cohort (n) | Count of observations with discrepancies (n) | The percent of discrepancies (%) |
| *Out of range values* | | | | |
| Demographic data elements | | 1,382,487 | 0 | 0 |
| Observation data elements | | 12,189,066 | 2,807 | 0.02 |
| Valid laboratory values | | 32,082,249 | 460,737 | 1.4 |
| *Incompatibility* | | | | |
| Age and diagnosis | | 72,168,619 | 5,303 | 0.01 |
| Age and procedure | | 39,453,903 | 49,366 | 0.13 |
| Gender and diagnosis | | 4,814,969 | 5,372 | 0.1 |
| Gender and procedure | | 1,350,001 | 1,136 | 0.1 |
| Drug and diagnosis | | 7,075 | 105 | 1.5 |
| Drug and drug interaction | | 41,775,300 | 10,592 | 0.03 |
| Inpatient only procedure | | 48,849,848 | 6,451 | 0.01 |
| Diagnosis and laboratory | | 64,877,882 | 242,932 | 0.4 |
| *Incompleteness* | | | | |
| Drug and laboratory | | 21,494,479 | 138,852 | 0.6 |
| Drug and continuous procedure | | 3,058,815 | 20,654 | 0.8 |
| Drug monitoring | | 5,553,985 | 2,208,587 | 39.8 |
| *Date and time error* | | | | |
| Laboratory time | | 147,810 | NA | NA |
| Date in future | Death date | 35,771 | 0 | 0.0 |
|  | Birth date | 1,382,487 | 0 | 0.0 |
|  | Medicine administration date | 33,755,873 | 0 | 0.0 |
|  | Procedure date | 77,126,809 | 0 | 0.0 |
| *Duplication* | | | | |
| Duplication | | 77,126,809 | 0 | 0.0 |
| Site L | |  |  |  |
| Template name | | Count of observations in the whole cohort (n) | Count of observations with discrepancies (n) | The percent of discrepancies (%) |
| *Out of range values* | | | | |
| Demographic data elements | | 5,035,940 | 0 | 0 |
| Observation data elements | | 293,624,431 | 53,294 | 0.02 |
| Valid laboratory values | | 134,298,393 | 9,867,356 | 7.4 |
| *Incompatibility* | | | | |
| Age and diagnosis | | 50,484,324 | 19,741 | 0.04 |
| Age and procedure | | 39,453,903 | 49,366 | 0.1 |
| Gender and diagnosis | | 3,477,777 | 5,363 | 0.2 |
| Gender and procedure | | 722,118 | 255 | 0.03 |
| Drug and diagnosis | | 7,268 | 17 | 0.2 |
| Drug and drug interaction | | 129,354,593 | 46,525 | 0.04 |
| Inpatient only procedure | | 42,703,255 | 125,855 | 0.3 |
| Diagnosis and laboratory | | 39,822,350 | 748,635 | 1.9 |
| *Incompleteness* | | | | |
| Drug and laboratory | | 63,208,864 | 283,639 | 0.4 |
| Drug and continuous procedure | | 2,203,071 | 48,063 | 2.2 |
| Drug monitoring | | 37,013,884 | 12,163,167 | 32.9 |
| *Date and time error* | | | | |
| Laboratory time | | 134,306 | NA | NA |
| Date in future | Death date | 169,948 | 0 | 0.0 |
|  | Birth date | 5,034,940 | 0 | 0.0 |
|  | Medicine administration date | NA | NA | NA |
|  | Procedure date | 53,927,301 | 0 | 0.0 |
| *Duplication* | | | | |
| Duplication | | 53,927,301 | 0 | 0.0 |
| Site M | |  |  |  |
| Template name | | Count of observations in the whole cohort (n) | Count of observations with discrepancies (n) | The percent of discrepancies (%) |
| *Out of range values* | | | | |
| Demographic data elements | | 1,258,443 | 0 | 0 |
| Observation data elements | | 60,000,000 | 725 | 0.001 |
| Valid laboratory values | | 6,409,857 | 69,062 | 1.1 |
| *Incompatibility* | | | | |
| Age and diagnosis | | 81,765,761 | 9,099 | 0.01 |
| Age and procedure | | 39,453,903 | 49,366 | 0.13 |
| Gender and diagnosis | | 6,027,933 | 35,604 | 0.6 |
| Gender and procedure | | 1,210,535 | 817 | 0.1 |
| Drug and diagnosis | | 2,990 | 11 | 0.4 |
| Drug and drug interaction | | 13,778,010 | 2,274 | 0.02 |
| Inpatient only procedure | | 68,449,729 | 9,088 | 0.01 |
| Diagnosis and laboratory | | 26,947,911 | 1.210.576 | 4.5 |
| *Incompleteness* | | | | |
| Drug and laboratory | | 8,990,775 | 237,345 | 2.6 |
| Drug and continuous procedure | | 4,524,661 | 8,157 | 0.2 |
| Drug monitoring | | 543,212 | 148,246 | 27.3 |
| *Date and time error* | | | | |
| Laboratory time | | 10,489 | 6,824 | 65.1 |
| Date in future | Death date | 84,654 | 0 | 0.0 |
|  | Birth date | 1,258,443 | 0 | 0.0 |
|  | Medicine administration date | 92,001,100 | 0 | 0.0 |
|  | Procedure date | 129,698,869 | 3 | 0.0000002 |
| *Duplication* | | | | |
| Duplication | | 129,698,869 | 0 | 0.0 |

^*^NA; data element needed to run the rule is not available in the database.
